# Short inversion time on delayed-enhancement magnetic resonance improves diagnostic accuracy in recent-onset heart failure

**DOI:** 10.1186/1532-429X-14-S1-P188

**Published:** 2012-02-01

**Authors:** Lorenzo Monti, Barbara Nardi, Veronica Lisignoli, Antonio Spinillo, Luca Balzarini

**Affiliations:** 1 Istituto Clinico Humanitas, Rozzano(MI), Italy; 2 Radiology, Istituto Clinico Humanitas, Rozzano(MI), Italy

## Summary

We evaluated the diagnostic consistency of a short inversion time (TI) Late Gadolinium Enhancement (LGE) sequence in the diagnostic work-up of recent onset heart failure of ischemic aetiology

## Background

Subendocardial LGE is a marker of ischemic aetiology of left ventricular dysfunction, and of viable myocardium. Using a high relaxivity contrast media such as gadobenate dimeglumine, however, little areas of subendocardial LGE can be masked by the high signal of the blood pool. We halved the optimal TI to null the signal of viable myocardium to obtain LGE images with high signal from viable myocardium, intermediate signal from the blood pool, and no signal from LGE areas, thus enhancing the presence of “hidden areas” subendocardial enhancement.

## Methods

23 patients with recent-onset heart failure (62 +/-11 years) were studied with CMR after contrast media administration ( MultiHance, Bracco) using LGE and short TI LGE sequences. All patients underwent invasive coronary angiography. Images were analyzed by an experienced (> 8 years of CMR reading)and 2 less experiences ( <18 months of CMR readings) readers, searching for the presence of areas of subendocardial enhancement.

## Results

17 patients had critical coronary artery disease at angiography. No one of the remaining 6 patients showed subendocardial enhancement. Of the 17 truly ischemic patients, 8 had areas > 25% subendocardial LGE, detected by all readers. In the 9 pts with <25% subendocardial LGE, a correct diagnosis was made in 8/9 by experienced reader and 6/9 by less experienced readers using normal TI. Short TI allowed all readers to detect < 25% subendocardial fibrosis in 9/9 patients.

## Conclusions

Short TI LGE significantly improves the diagnostic accuracy for ischemic aetiology of heart failure, with a greater improvement in less experienced readers. In our small series of ischemic patients without transmural myocardial infarction, short TI LGE allowed to detect subendocardial fibrosis also in patients "without" subendocardial LGE.

## Funding

None.

**Figure 1:**
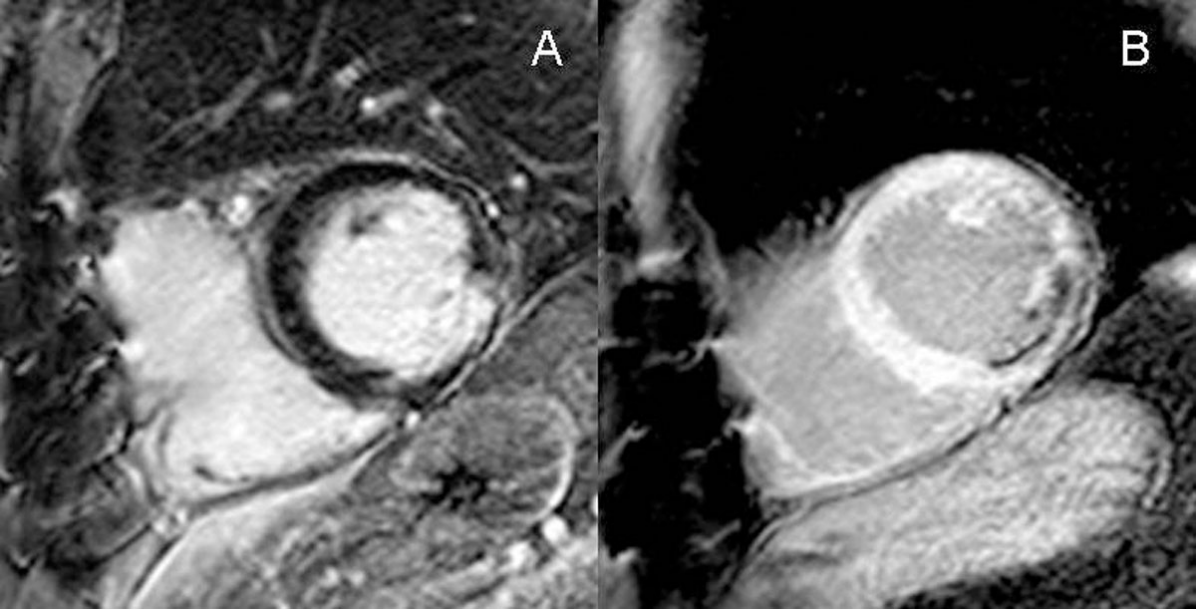
Subendocardial inferior scar. Short TI LGE (Panel B) allows a more confident detection of the scar and unveils partial papillary muscle fibrosis.

